# The Role of Endothelins, IL-18, and NGAL in Kidney Hypothermic Machine Perfusion

**DOI:** 10.3390/biomedicines9040417

**Published:** 2021-04-13

**Authors:** Karol Tejchman, Adam Nowacki, Katarzyna Kotfis, Edyta Skwirczynska, Maciej Kotowski, Labib Zair, Marek Ostrowski, Jerzy Sienko

**Affiliations:** 1 Department of General and Transplantation Surgery, Pomeranian Medical University, 70-111 Szczecin, Poland; ktej78@pum.edu.pl (K.T.); maciej.j.kotowski@gmail.com (M.K.); labib@poczta.onet.pl (L.Z.); mostrowski@poczta.onet.pl (M.O.); jsien@poczta.onet.pl (J.S.); 2 Department of Vascular and General Surgery, Pomeranian Medical University, 70-111 Szczecin, Poland; itskorn@gmail.com; 3 Department of Anesthesiology, Intensive Therapy and Acute Intoxications, Pomeranian Medical University, 70-111 Szczecin, Poland; 4 Department of History of Medicine and Ethics, Pomeranian Medical University, 70-204 Szczecin, Poland; edytas@pum.edu.pl

**Keywords:** kidney transplantation, hypothermic machine perfusion, static cold storage, endothelin, IL-18, NGAL, LifePort, outcome

## Abstract

Ischemia-reperfusion injury (IRI) occurring after renal transplantation is a complex biochemical process that can be monitored by specific biomarkers. The roles of those are not yet fully elucidated. The aim of this study was to analyze the concentrations of endothelins (ET-1, ET-2, and ET-3), interleukin-18 (IL-18), and neutrophil gelatinase-associated lipocalin (NGAL) during the reperfusion of human kidneys grafted from brain dead donors and later transplanted. The study group (*n* = 44) was analyzed according to the method of kidney storage: Group 1 underwent hypothermic machine perfusion (HMP) in the LifePort perfusion pump (*n* = 22), and Group 2 underwent static cold storage (SCS) (*n* = 22). The analysis of kidney function was performed daily during the first seven days after transplantation. The kidneys in Group 1 were characterized by higher absolute concentrations of ET-1, IL-18, and NGAL, as well as a lower concentration of ET-2 (*p* = 0.017) and ET-3. The relative increase of ET-1 (*p* = 0.033), ET-2, and ET-3 during reperfusion was lower in this group, while the relative decrease of NGAL was higher. Group 1 was also characterized by significant decrease of IL-18 (*p* = 0.026) and a tendency for better kidney function based on the higher total diuresis, higher glomerular filtration rate (GFR), higher potassium level, lower serum creatinine, and lower urea concentration during the seven-day postoperative observation period. The long-term beneficial impact of hypothermic machine perfusion on the outcome of transplanted kidneys may rely on the early modified proceedings and intensity of ischemia-reperfusion injury reflected by the dynamics of the concentrations of examined biomarkers.

## 1. Introduction

Kidney transplantation (KTx) is the only curative treatment option for patients suffering from end-stage renal failure. The growing imbalance between the number of surgical procedures and patients on the transplant waiting lists remains a major concern in all countries. Despite the increasing use of donation after circulatory death (DCD) and extended criteria donor (ECD), overall patient and allograft survival rates are improving. The influence of donor condition on outcome is multifactorial [1], but organs with extended-criteria are known to be more susceptible to ischemia-reperfusion injury (IRI), which is a major determinant of delayed graft function (DGF) [2,3,4]. IRI consists of two phases: ischemia, when the blood flow is interrupted for a period of time separating organ procurement from transplantation resulting in cell energy depletion, and reperfusion, when the blood flow is restored resulting in oxidative stress, microcirculatory impairment, inflammation, and apoptosis [5,6,7]. Those two phases involve different organ responses, but the total damage is additive. In addition to immunological rejection, IRI is a major cause of graft loss and dysfunction in clinical transplantation [3,8]. The pathophysiology of this process is complex and multifactorial [9]. Numerous pathways have opened the field for therapies against certain points of interest, e.g., the impairment of endothelium relaxation, the scavenging of free-radicals, and the blockade of neutrophil activation and adhesion [2]. Advances in IRI studies regarding its molecular mechanisms have led to different strategies that are used to reduce the detrimental effects of IRI. One of the modifiers of the ischemic phase is the use of machine perfusion (MP), which is a well-established approach for decreasing the incidence of DGF and improving late outcomes, especially for DCD and ECD [10,11]. MP offers the advantage of preventing mitochondrial and tissue damage. In static cold storage (SCS), the massive accumulation of metabolites derived from anaerobic respiration during the ischemic phase increases the hazard caused by oxidative stress in reperfusion [12,13]. There are also promising experimental strategies of upgrading MP with oxygenation (hypothermic oxygenated perfusion: HOPE) or with an O_2_ carrier with anti-oxidant capacities (normothermic machine perfusion: NMP), resulting in improved kidney recovery and an enhanced late graft outcome [14].

Specific biomarkers can be used to identify IRI, e.g., endothelins (ET-1, ET-2, and ET-3), interleukin-18 (IL-18), and neutrophil gelatinase-associated lipocalin (NGAL) in conjunction with “classic” markers of kidney function like diuresis, glomerular filtration rate (GFR), creatinine, urea, and potassium. Endothelins are peptides released from kidney endothelial cells after four-to-seven minutes as a response to the injurious stimuli [15]. They are the most powerful endogenous chemicals that affect vascular tone across all organ systems and that play major roles in IRI [2,16,17]. IL-18 counts among proinflammatory cytokines that influences the proliferation, growth, and stimulation of immune response cells. Its level significantly increases in conditions related to kidney injury [18] and strongly induces the synthesis of interferon gamma (IFN-γ), along with strong inflammatory reactions and tissue damage in response to ischemia, thus correlating with an increased risk of graft insufficiency [19,20]. NGAL is considered to be an early marker of kidney injury that increases in response to inflammation or endothelium damage in renal tubules and results in the proliferation of new epithelial cells [21,22].

We hypothesized that MP improves the late outcome of kidney transplantation. There might be a link between the method of kidney storage and both modern and “classic” early markers of kidney function. Therefore, the aim of this study was to compare two groups of recipients in relation to the method of kidney storage: Group 1 underwent hypothermic machine perfusion (HMP) in LifePort, and Group 2 underwent SCS.

## 2. Materials and Methods

### 2.1. Study Group and Inclusion/Exclusion Criteria

**Figure 1:**
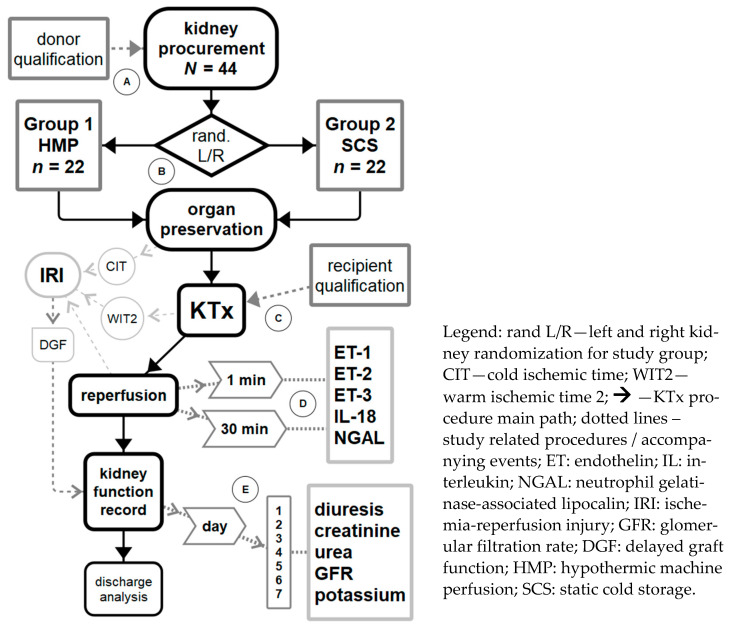
Study flowchart. (**A**) Donor qualification procedure and donor parameters record. (**B**) Randomization for study group and preservation. (**C**) Recipient qualification with parameters record and kidney transplantation (KTx) surgical procedure. (**D**) Blood samples for biomarkers during reperfusion. (**E**) Seven-day daily repeated 24 h urine collection and blood samples for kidney function evaluation.

Study procedures were summarized in Figure 1 (study flow chart). Kidney donors were qualified for the study after standard procedure for clinical assessment of brain death (DBD) according to standard criteria (SCD). Organ procurement only regarded a pair of kidneys. Multiorgan donations and cases of single kidney procurement were excluded from the study. The standard surgical procedure comprised the following steps: laparotomy with thoracotomy, aorta and vena cava inferior cannulation, aorta ligation in abdominal segment, intracorporeal organ flushing with a 4 °C Custodiol HTK (histidine–tryptophan–ketoglutarate) solution (Custodiol® HTK, Essential Pharmaceuticals, LLC, 1009 Slater Road 210B, Durham, NC 27703, USA), and nephrectomy on both sides with proper tissue margin for storage and transplantation purposes (Figure 1A). Multiorgan donations were excluded because organs were flushed with different, University of Wisconsin (UW)-based, perfusion solutions: CoStorSol™ and SPS-1®. For the purpose of the study, only a pair of kidneys was selected and only one type of preservation solution was used. The composition of Custodiol HTK was: 15.0 mmol/L sodium chloride, 9.0 mmol/L potassium chloride, 1.0 mmol/L potassium hydrogen 2-ketoglutarate, 4.0 mmol/L magnesium chloride hexahydrate, 18.0 mmol/L histidine HCI H_2_O, 180.0 mmol/L histidine, 2.0 mmol/L tryptophan, 30.0 mmol/L mannitol, 0.015 mmol/L calcium chloride 2 H_2_O, sterile water for injection, and a 50 mEq Cl^−^ anion. The physical properties of the fluid were as follows: pH of 7.02–7.20 at 25 °C (77 °F) (pH 7.4–7.45 at 4 °C (39.2 °F)) and an osmolality of 310 mOsm/kg.

One kidney from the pair was randomly inserted into the HMP pump (LifePort^®^, Organ Recovery Systems; Itasca, Illinois) and assigned to Group 1, whereas the other one was inserted into standardized container for SCS and assigned to Group 2. This resulted in 22 pairs of kidneys (*n* = 44) randomized to equal groups (Figure 1B). The kidney from Group 1 was placed in LifePort directly after procurement. Systolic pressure was set to 30 mmHg as a default value according to the manufacturer’s manual, with the indication that if the flow did not reach 100 mL/min, the pressure had to be raised to 35 mmHg and then to a maximal value 40 mmHg. If the flow could not reach 100 mL/min, then all LifePort and kidney connections had to be checked. In this group, there was only 1 case when the pressure had to be raised to 35 mmHg for 2 h. The flow was stabilized, and the pressure was set to default like with all other cases to 30 mmHg. The mean flow was 208.4 ± 49.0 mL/min (43–232 mL/min). All kidneys had flow stabilized over 200 mL/min after 3 h without changing the pressure except for the above-mentioned case. The mean resistance was 0.14 ± 0.08 mmHg/mL/min (0.1–0.6 mmHg/mL/mi), mean temperature 3.1 °C (2.7–3.4 °C). All parameters were monitored and recorded during the entire preservation period at least once every hour. Kidney containers (product code: H-112 Large kidney container) for Group 2 (SCS) were provided by Medans Oy (Pihatörmä 1 A, 3. Krs, 02240 ESPOO, FINLAND). After randomization, one kidney was placed in the container filled with the preservation solution. The containers were placed in a special transportation box made from thermal insulation material that held ice in the melting temperature and providing 2–4 °C in the chamber. The temperature was maintained, monitored, and recorded during the entire preservation period. Both LifePort and SCS containers were transported to the transplantation department at the same time.

The procedure of kidney allocation was conducted according to standards governed by the National Transplantation Committee in Poland (Poltransplant). The first recipient received a kidney from SCS, and the second received a kidney from HMP. During the study period, only one perfusion machine was available on site. Due to the known influence of the storage method on DGF incidence, it was not ethically justified to use HMP kidney before SCS for the purpose of randomization.

### 2.2. Transplant Procedure

All kidney recipients were admitted to the nephrology department of a university transplant center and qualified according to medical examination. The following data were recorded: hemodialysis time, serum creatinine, urea, estimate of the glomerular filtration rate (eGFR), and blood morphology (Figure 1). The following KTx procedure was performed at the surgical department according to a standard protocol with the following steps: approach to retroperitoneal space (preferably on the right side), preparation of external iliac vessels, anastomosis of renal vessels end-to-side, reperfusion, hemostasis, anastomosis of the ureter to the bladder, and closure. The collection of blood samples during reperfusion is described in Section 2.4. Laboratory analysis.

### 2.3. Post-Transplant Care

After kidney transplantation, the recipients were hospitalized in a surgical transplant department for 7 days. During that period, patients were monitored according to the standard postoperative procedure. Classic markers of kidney function were measured every day, including diuresis (24-h urine collection) and plasma concentrations of creatinine, urea, and potassium. The GFR was estimated using the Cockcroft–Gault equation (eGFR) according to standard laboratory assay methods (Figure 1E). Postoperative observation was complemented with a DGF incidence record. Delayed graft function, resulting directly from IRI, was recorded during the 7-day hospitalization and defined as the need to use of dialysis in the first postoperative week. Afterwards, recipients were transferred to the nephrology department for further post-transplant care.

### 2.4. Laboratory Analysis

#### 2.4.1. Sample Collection and Laboratory Kits

After restoring the circulation to the transplanted kidney, two 15-mL samples of blood were obtained, the first at the 1st minute after reperfusion and the second at 30 min after reperfusion (Figure 1D). Blood was collected via venipuncture of the renal vein, poured into ethylenediaminetetraacetic (EDTA) tubes, stored at 4 °C, and promptly delivered to the University Central Laboratory within working hours for assay. The concentrations of biomarkers were measured using an ELISA. Endothelins were measured using ELISA kits by BioSupply Ltd., 11 The Grove, Shipley, BD18 4LD. “ENDOTHELIN-1 (1–31) ELISA KIT”-wells bound with ET-1 antigen^25–31^ rabbit immunoglobulin (IgG) affinity purify and horseradish peroxidase (HRP) conjugated to anti-human ET-1 (1–31) antibody had a minimum sensitivity detection limit of 0.62 pg/mL and a dynamic range of 1.56–200 pg/mL. “ENDOTHELIN-2 (1–31) ELISA KIT”-wells coated with ET-2 antigen^25–31^ rabbit IgG affinity purify and HRP conjugated with human ET-2 (1–31) antibody had a minimum sensitivity detection limit of 4.0 pg/m4l and a dynamic range of 3.91–500.0 pg/mL. “ENDOTHELIN-3 ELISA KIT”-wells bound with ET-3 antigen 15–21 rabbit IgG affinity purify and HRP conjugated to endothelin-3 antibody had a detection limit of 0.36 pg/mL. We also used the “Human Total IL-18 DuoSet ELISA Kit” (R&D Systems Inc., Minneapolis, MN, USA), with an assay range of 11.7–750 pg/mL, and the “Human Lipocalin-2/NGAL Quantikine ELISA Kit” (R&D Systems Inc, Minneapolis, MN, USA), with an assay range of 0.2–10 ng/mL. All technical ELISA procedures were performed according to kit manufacturer manuals.

#### 2.4.2. General Overview of ELISA Procedure Performed

An antibody specific for a specific biomarker was pre-coated onto a microplate. Standards and samples were pipetted into the wells, and any biomarker present was bound by the immobilized antibody. Following incubation, unbound samples were removed during a wash step, and then a detection antibody specific for the certain biomarker was added to the wells and bound to the combination of capture antibody-biomarker in the sample. Following a washing procedure aimed at removing any unbound combination, an enzyme conjugate was added to the wells. Following the incubation and wash steps, a substrate was added. A colored product, tetramethylbenzidine (TMB), was formed in proportion to the amount of biomarker present in the sample. The reaction was terminated by the addition of acid, and the absorbance was measured. A standard curve was prepared from seven biomarker standard dilutions, and the biomarker sample concentration was determined.

#### 2.4.3. Detailed ELISA Procedure and Results Reading

Plasma was collected using EDTA as an anticoagulant and centrifuged for 15 min at 1000× *g* at 2–8 °C. Samples were pre-tested to determine the dilution factor. All reagents were brought to room temperature. A standard/sample diluent of 1.0 mL was added into freeze-dried standard and sit for a minimum of 15 min prior to making dilutions (16,000 pg/mL). Eppendorf (EP) tubes containing diluent were prepared with a dilution series to produce the recommended concentration for a standard curve: 2000, 1000, 500, 250, 125, 62.5, and 31.2 pg/mL. The biotin–conjugate antibody diluent (1:100), the streptavidin–HRP diluent (1:100), and the wash buffer with deionized water (1:30) were diluted before use. Additionally, 100 μL of standards and test samples were added to each well and incubated for 1 h at 37 °C, and 100 μL of a biotin–conjugate antibody solution was added, incubated for 1 h at 37 °C, and washed 3 times. Next, 100 μL of a streptavidin–HRP solution was added, incubated for 30 min at 37 °C, and washed 5 times. Then, a 90 μL substrate solution was added and incubated for 15–20 min at 37 °C under a dark condition. Finally, a 50 μL stop solution was added. Optical density was detected within 5 min under 450 nm using a Microplate Reader ELX 808IU spectrophotometer (Bio-Tek Instruments Inc.). Each standard, control, and sample were duplicated and averaged, and then they were subtracted with the average zero standard optical density. The data were analyzed using software capable of generating a log/log curve fit. Concentrations were read from the standard curve and multiplied by the proper dilution factor.

### 2.5. Ethical Approcal

This study received approval of the Bioethical Committee of the Pomeranian Medical University in Szczecin (No. KB-0012/19/13, dated 21st January 2013).

### 2.6. Statistical Analysis

Statistical analysis was performed using software Statistica 13 (TIBCO Software Inc., 3307 Hillview Avenue, Palo Alto, CA 94304, USA). Data distribution was checked with Shapiro–Wilk test and confirmed with the Kolmogorov–Smirnov/Lilliefors test—which involved the implementation of descriptive statistics, medians, quartiles and non-parametric tests—that revealed that it was different than normal for most variables. Dependent variables were all numeric absolute and relative measurements recorded in the study. Grouping variables were assignments to examined Groups 1 and 2. The following 2-group non-parametric independent samples tests were used: Mann–Whitney U, 2-group Kolmogorov–Smirnov, and Wald–Wolfowitz runs (see Section 3.1). The 7-day repeated measurements were analyzed with Friedman ANOVA and Kendall’s W (coefficient of concordance) tests. The analysis in both examined groups was performed using the effective hypothesis decomposition of two-way ANOVA for the effect of the storage method. The data standardization for relative classic markers comparisons was performed with Statistica 13 (see Section 3.3). Interactions between new and classic biomarkers were analyzed using Spearman’s rank-order correlation, Goodman and Kruskal’s gamma, and Kendall rank correlation coefficient (see Section 3.4). Statistical significance was set at *p* < 0.05.

## 3. Results

### 3.1. Study Group Characteristics

The characteristics of recipients qualified for kidney transplantation are presented in Table 1. There were no significant statistical differences between both groups (*p* > 0.05).

The median age of kidney recipients was 55 years in Group 1 vs. 54 years in Group 2. There were 15 men and 7 women in Group 1, and there were 11 men and 11 women in Group 2. The hemodialysis times before KTx were 37 vs. 48 months in Group 1 vs. 2. The median serum urea values were 101.0 vs. 85.5 mg/dL, the median serum creatinine values were 7.4 vs. 7.9 mg/dL, the median eGFR values were 7.7 vs. 6.0 mL/min/1.73 m^2^, the median leukocyte values were 7.7 vs. 6.5 G/μL, the median platelet values were 210 vs. 194 G/μL, the median potassium values were 4.5 vs. 4.6 mmol/L, and the median hematocrit values were 37.7 vs. 35.3 L/L for Group 1 vs. 2, respectively. The median mismatch number of human leukocyte antigens A, B, and DR in both groups was 1. According to the national kidney transplant allocation rules there are preferential points calculated according to the number of matching human leukocyte antigen (HLAs): +2 points per 1 matching HLA-A, +5 points per 1 matching HLA-B, and +10 points per 1 matching HLA-DR. Points were added to the overall score calculated based on other qualification parameters. The median points were 20 in Group 1 and 17 in Group 2. There were two cases of DGF in Group 1 (9.1%) and three cases in Group 2 (13.6%). Organ preservation time measured by cold ischemic time (CIT) ranged from 4.5 to 26.9 h (median: 18.4 h) in Group 1 and 4.9–22.9 h (median: 11.6) in Group 2. The immunosuppression treatment protocol for all recipients included a calcineurin inhibitor (tacrolimus (Tac)/cyclosporine (CsA)), antimetabolite (mycophenolate mofetil: MMF), and steroid. Overall, 21 recipients received Tac and one received cyclosporine CsA in both groups. All recipients received MMF, and all of the patients received steroids in both groups.

### 3.2. Absolute Serum Concentrations of ET-1, ET-2, ET-3, IL-18, and NGAL in the 1st and 30th Minutes of Kidney Reperfusion

**Figure 2:**
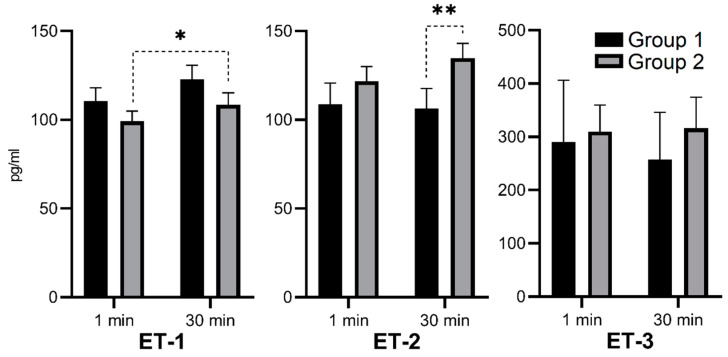
Median serum concentration of ET-1, ET-2, and ET-3 in the 1st and 30th minutes of reperfusion in storage groups. (* *p* = 0.033; ** *p* = 0.017).

**Figure 3:**
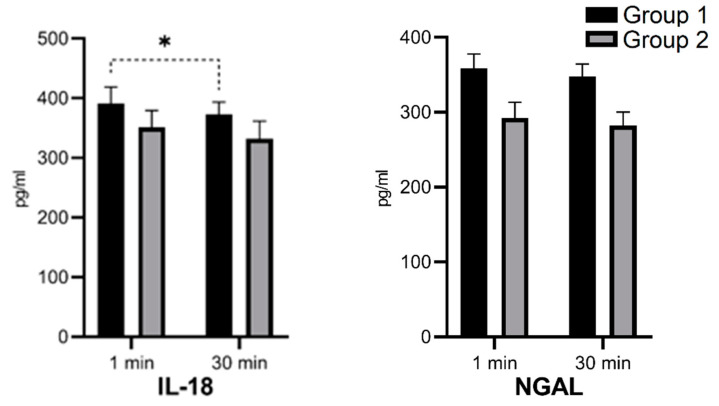
Median serum concentration of IL-18 and NGAL in 1st and 30th minutes of reperfusion in storage groups. (* *p* = 0.026).

Group 1 was characterized by higher median concentrations of ET-1, IL-18, and NGAL and lower concentrations of ET-2 and ET-3. This tendency was similar at 1 and 30 min after reperfusion. The ET-2 median concentration in the 30th minute of reperfusion was significantly lower in Group 1 (Figure 2; ** *p* = 0.017). After 30 min of reperfusion, there was an increase of the median ET-1 concentration in both groups—in Group 2, it was statistically significant (Figure 2; * *p* = 0.033). After 30 min of reperfusion, there was a decrease of median IL-18 concentrations in both groups—in Group 1, it was statistically significant (Figure 3; *p* = 0.026). After 30 min of reperfusion, there was a tendency towards a decrease of the median NGAL concentrations in both groups (Figure 3). ET-2 and ET-3 median concentrations after 30 min of reperfusion revealed a tendency to a decrease in Group 1 and a tendency to increase Group 2, but statistical significance was not met and an impact on the plots was limited, respectively (Figure 2).

### 3.3. Relative Serum Concentration Changes (Δ) of ET-1, ET-2, ET-3, IL-18, and NGAL After 30 Min of a Kidney Reperfusion

**Figure 4:**
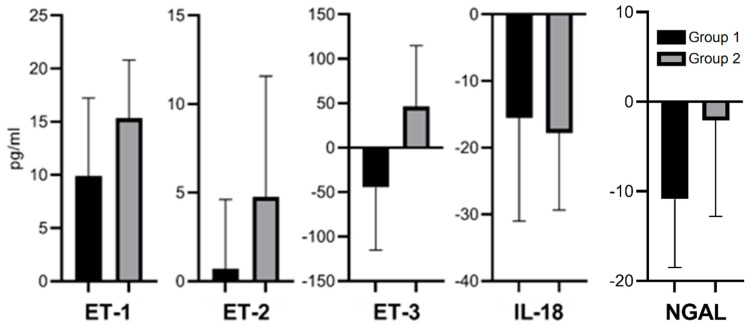
Median of serum concentration changes (Δ) between 1st and 30th minutes of the reperfusion of ET-1 (*p* = 0.5494), ET-2 (*p* = 0.1848), ET-3 (*p* = 0.1300), IL-18 (*p* = 0.7160), and NGAL (*p* = 0.4688).

Two additional variables were calculated based on available concentrations: the first one was the subtraction result of concentrations in the 30th and 1st minute, appointed as Δ, and the second one was the new median for the above mentioned variable. These results enabled us to present the median of differences instead of the differences of medians (Figure 4). Since the distribution of data regarding concentrations was different than normal, such an approach allowed us to further analyze an additional variable independent of the absolute concentration and with its own quartiles. This was also analyzed in Results 3.5. We assumed that the change in concentrations might played a more important role than the absolute values. Differences between Groups 1 and 2 in biomarker concentration change (Δ) were statistically insignificant, but we noticed a tendency towards greater Δ increases of ET-1, ET-2, and ET-3 in Group 2. There was a tendency towards greater Δ decrease of NGAL in Group 1, and a tendency towards greater Δ decrease of ET-3 in Group 2.

### 3.4. Classic Biomarkers of Kidney Function During the Seven-Day Postoperative Observation Period

**Figure 5:**
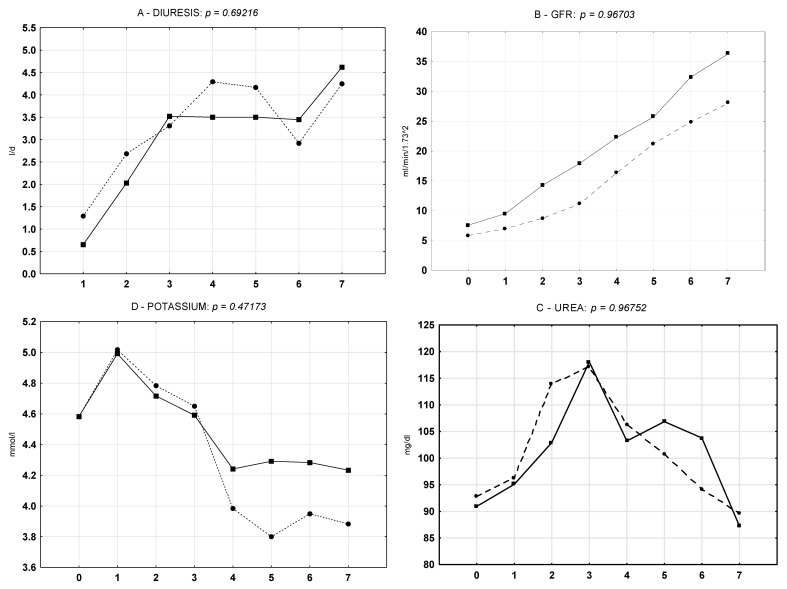
Weighted arithmetic means of kidney function parameters during 7-day postoperative observation in storage groups (▉ Group 1, ⚫ Group 2) (differences within group, *p* < 0.001). Effective hypothesis decomposition of two-way ANOVA test for the effect of storage method (differences between groups, *p* > 0.05).

**Figure 6:**
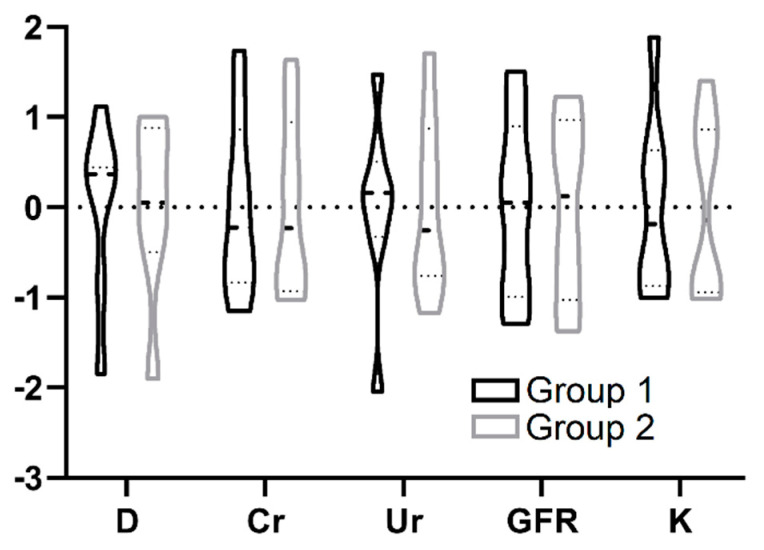
Distribution of standardized kidney function parameters: diuresis (D), creatinine (Cr), urea (Ur), GFR, and potassium (K) during the 7-day postoperative observation in storage groups.

For seven days after KTx, both groups were characterized by a significant increase of diuresis and GFR, along with decreases of serum concentration of urea, potassium (Figure 5), and creatinine (not shown). The analysis was performed using Friedman ANOVA and Kendall’s W tests. Results were statistically significant for every parameter in the whole examined group, as well as separately in Groups 1 and 2 (*p* < 0.001). We observed a tendency for higher GFR over seven days of observation in Group 1, as well as a tendency for higher diuresis, GFR, and potassium at day seven (*p* > 0.05) (Figure 5A,B,D). Afterwards, the data were analyzed by the effective hypothesis decomposition of the two-way ANOVA test for the statistic effect of storage method. There were no significant differences in the characteristics or dynamics of kidney function parameter changes during the seven-day observation period between Groups 1 and 2 (*p* > 0.05). The plot lines in Figure 5 linking weighted arithmetic means show a tendency for a higher mean GFR and a higher potassium from day four in Group 1 (*p* = 0.96703 and *p* = 0.47173, respectively), but statistical significance was not met. Finally, all of the above data were summarized on a single violin plot that showed the distribution of standardized kidney function parameters within seven days. The result aggregation, represented by a wider plot, revealed a slight tendency towards higher diuresis (D) and higher urea concentration (Ur) in Group 1 during postoperative observation. Other parameters were characterized by a similar median and distribution (Figure 6).

### 3.5. Correlations Between Absolute and Relative Concentrations of ET-1, ET-2, ET-3, IL-18, and NGAL and Kidney Function Parameters During Seven-Day Postoperative Observation

All biomarker concentrations measured during reperfusion time (ET-1, ET-2, ET-3, IL-18, and NGAL) were correlated with kidney function parameters during the seven-day observation period (diuresis, creatinine, urea, GFR, and potassium). The following relative changes (Δ) were included: (30 min)—(1 min) and (day 7)—(day 0/1). The results are summarized on a heatmap in Table 2. The intensity of colors is proportional to the number of statistically significant correlations between the corresponding variables. Columns and rows with no significant correlations were removed from the table. Improved kidney function was assigned to higher D and GFR, as well as to lower concentrations of Cr, U, and K. Better kidney function was marked with warm color gradient (red). Worse kidney function was assigned to the opposite alterations of the above-mentioned parameters and marked with a cold color gradient (blue). Empty and filled black dots were added as complementary information that was the mathematical result of correlation (empty—positive; filled—negative), but the following aggregation results refer to color as the impact on kidney function.

There was an aggregation of statistically significant correlations between a positive impact on kidney function and a higher ET-1 concentration in the first minute of reperfusion in Group 2 (Table 2*). Similar aggregation was not observed in Group 1. There was an aggregation of statistically significant correlations between a positive impact on kidney function and a higher ET-1 concentration in the 30th minute of reperfusion and its increase after 30 min (Δ) in Group 1 (Table 2**). A similar aggregation was not observed in Group 2. There was an aggregation of statistically significant correlations between a negative impact on kidney function and a higher ET-2 concentration in the 30th minute of reperfusion in both groups, but the aggregation was more intense in Group 1 (Table 2#). There was an aggregation of statistically significant correlations between a negative impact on kidney function and a higher ET-3 concentration in the 30th minute of reperfusion in Group 2 (Table 2##). There was an aggregation of statistically significant correlations between a negative impact on kidney function and higher value of IL-18 (Δ) during reperfusion in Group 1 (Table 2+). A similar aggregation was not observed in Group 2. Since IL-18 was decreasing during reperfusion (Figure 4), it mathematically translated into the link that a greater decrease of IL-18 in Group 1 during reperfusion correlated with better kidney function parameters, except for potassium (Table 2+). There were single statistically significant correlations between a negative impact on kidney function and higher NGAL concentrations in Group 1 (Table 2++)

## 4. Discussion

The results of our study showed that kidneys in the hypothermic machine perfusion compared with static cold storage were characterized by higher absolute concentrations of ET-1, IL-18, and NGAL, as well as lower concentrations of ET-2 and ET-3. The relative increase of ET-1, ET-2, and ET-3 during reperfusion was lower in this group, while the relative decrease of NGAL was higher. HMP was also characterized by a significant decrease in the IL-18 level and a tendency for better kidney function based on higher total diuresis, GFR, potassium level, lower creatinine, and urea during a postoperative seven-day observation period. We further discuss the relevance of these findings within each cytokine subgroup.

### 4.1. Endothelins

The analysis of serum concentrations of ET-1, ET-2, ET-3, IL-18, and NGAL revealed a tendency of higher ET-1, IL-18, and NGAL concentrations, as well as lower ET-2 and ET-3 concentrations, in Group 1 versus Group 2. Only the ET-2 difference was statistically significant, but we observed consistent characteristics at the 1st and 30th minutes after reperfusion. It was demonstrated in animal models that ET-1 caused an increase of vascular resistance, a decrease of renal blood flow, and a decrease of the glomerular filtration rate [23,24]. ET-1 increases the permeability of glomerular vessels inducing proteinuria. It also activates the phospholipase A2 of mesangial cells and releases thromboxane A2, which, in turn, increases interstitial proliferation and glomerular hyalinization [25,26]. An increased serum concentration of ET 1 after kidney transplantation might be considered a negative risk factor, predicting increased vascular resistance and specific complications incidence connected with a pathologic background of DGF. In our study, there was a statistically significant increase of the ET-1 concentration in Group 2 during the 30 min of reperfusion (Figure 2), but it was hard to determine whether absolute concentrations played a role or if it had an increased release during reperfusion. Since ET-1 is synthetized by the kidney endothelium, vessel plain muscles, and mesangial cells (as well as blood cells during inflammation [27]), we hypothesized that an early release (4–7 min) of ET-1, confirmed in some studies [23], may be a response to the injurious stimulus to kidney endothelial cells. Therefore, the induction of ET-1 synthesis may be triggered during the reperfusion phase of IRI. By affecting vascular tone across organ systems, ET-1 is involved in cardiovascular function, fluid-electrolyte homeostasis, and neuronal mechanisms across diverse cell types [15,28,29]. There are at least four known endothelin receptors (ET_A_, ET_B1_, ET_B2_, and ET_C_), all of which are G protein-coupled receptors whose activation results in an elevation of intracellular-free calcium level [30]. The ET_A_ receptor for ET-1 is primarily located on vascular smooth muscle cells, mediating vasoconstriction, whereas the ET_B_ receptor for ET-1 is mainly located on endothelial cells, causing vasodilation due to nitric oxide (NO) release [31]. Since NO plays an important role during reperfusion [32], we hypothesized that ET_A_ receptor stimulation and subsequent vasoconstriction may not be an exclusive mechanism of ET-1 activity. In our study, we observed a significantly higher concentration of ET-2 after 30 min of reperfusion in Group 2 compared with that of Group 1. The ET-2 has an affinity for the same receptors as ET-1. Studies on ET-2 in kidney physiology and pathology have revealed that ET-2 concentration increases in the condition of oxidative stress and may be the indicator of renal dysfunction [33]. The expression of endothelin receptors in renal blood vessels (small and intermediate arteries) seems to be important in the diagnosis of damage during acute tubular necrosis and antibody-mediated rejection [34]. In our study, the ET-2 tended to be more expressed in Group 2. After 30 min of reperfusion, ET-2 concentrations were significantly higher in Group 2, suggesting the development of more advanced ischemia induced changes in comparison to Group 1. The ET-3 infusion in humans causes the induction of vasoconstriction through activation of the ET_B2_ receptor [35], and in rats, it causes significant hemodynamic changes in kidneys with a GFR decease [36]. In our study, 30 min of reperfusion had an opposite influence on the ET-3 concentration. It was decreased in Group 1 but increased in Group 2, which may also suggest more intense degenerative and injury changes in the SCS kidney.

### 4.2. Interleukin-18 (IL-18)

The basis of our hypothesis was that IL-18 expression and blood secretion may vary between examined groups and may correlate with classic kidney function parameters. In our study, we observed a statistically significant decrease in IL-18 concentration in Group 1 after 30 min of reperfusion. A significant decrease in serum IL-18 concentration may suggest a better initial kidney condition after HMP storage.

The IL-18 is a proinflammatory cytokine produced by antigen-presenting cells (APCs), and it modulates immune deviation [37]. The induction of IFN-γ synthesis correlates with kidney injury and is associated with a risk of graft insufficiency [18,19,20,38]. IL-18 is well-documented as being involved in various types of kidney diseases [39]. Studies have suggested that IL-18 with NGAL and creatinine are the most sensitive markers of DGF [40]. Moreover, IL-18 positively correlates with kidney function at 6 and 24 months after transplantation [41]. Urine-detected IL-18 is an early diagnostic marker of acute kidney injury (AKI), and it precedes symptoms by 24–48 h [42]. Animal models have shown that mice deficient in IL-18 or those administered antibodies neutralizing anti-IL-18 are resistant to acute kidney disease induced by ischemia/reperfusion [43]. Additionally, IL-18 seems to be associated with allograft rejection and may be a useful marker of its risk in renal transplant recipients [44].

### 4.3. Neutrophil Gelatinase-Associated Lipocalin (NGAL)

We hypothesized that HMP resulting in less kidney damage would correlate with smaller NGAL concentrations or its decrease during reperfusion. In our study, we observed a tendency for higher median decrease of NGAL in Group 1, which might suggest a less expressed graft injury, but we also observed higher absolute concentrations of NGAL in Group 1.

NGAL is expressed by tubular epithelial cells as a response to inflammation [18,20], injury, and tubulointerstitial damage [45]. It is thought to be an early, noninvasive marker of kidney injury, which can be detected before fully blown disease [46,47]. NGAL earned a position as a valuable marker for monitoring patients after transplantation for its predictive value towards DGF incidence [40,48,49]. The level of NGAL expression seems to be associated with the degree of kidney dysfunction and may help to indicate patients who are at a higher risk of faster decline of kidney function [50].

### 4.4. Kidney Function and HMP

The seven-day analysis confirmed a significant increase in diuresis and GFR, together with a decrease of creatinine, urea, and potassium in both examined groups. The detailed ANOVA calculation in the study groups revealed a tendency towards higher GFR during hospitalization in Group 1, as well as a tendency for higher diuresis, GFR, and potassium at day seven in Group 1. Despite statistically significant improvement of kidney function parameters during the seven-day observation period, the dynamics of those changes were similar in both groups. We believe that a larger study group might show a significance of the above-mentioned tendencies, but studies tend to stress a relation between HMP and a long-term outcome. For this study, we only selected kidney donations flushed with one type of preservation fluid (Custodiol HTK), so we believe that we eliminated this factor as a source of potential differences.

Systematic reviews, meta-analyses, randomized controlled trials, and other studies have been coherent in showing that HMP allows for a longer safe cold ischemic time, improved cellular metabolism, and improved perfusion parameters due to the protective effects on kidney epithelium [51,52,53,54,55]. HMP decreases the DGF rate for both DBD and DCD kidneys [56,57]. Systematic reviews of allograft function, graft and patient survival, acute rejection, and parameters of tubular, glomerular, and endothelial function have revealed that HMP improves renal preservation through the better maintenance of tubular, glomerular, and endothelial function and integrity, especially in short-term outcomes after renal transplantation [58]. HMP seems to improve the graft survival at three years, but long-term outcomes still need to be further investigated [59].

### 4.5. Modern vs. Classic Kidney Function Biomarkers

In clinical practice, kidney damage is generally detected by changes in serum creatinine and a creatinine-based eGFR, as well as protein excretion. A significant increase in serum creatinine concentration indicates that more than 50% of the glomerular function has been lost or dysfunctional [60]. Tubular dysfunction after kidney storage and transplantation is a source of elevated urea and potassium, but it may also be influenced by both rejection episodes and toxic effects of calcineurin inhibitors [61]. Diuresis itself remains the most easily obtainable clinical parameter of allograft recovery and may be used to construct multi-variable models and nomograms, thus allowing for the prediction of urine output after kidney transplantation [62]. Nonetheless, these models are often insensitive or not specific, lack accuracy, and can only be used late in the disease. On the other hand, modern biomarkers that meet the definition of the National Institutes of Health Biomarker Definition Working Group—“A characteristic that is objectively measured and evaluated as an indicator of normal biological processes, pathogenic responses, or pharmacological responses to a therapeutic intervention”—should be detected before irreversible damage occurs. Using the WHO definition, they can be measured in the body or its products and influence, or they can predict the incidence of outcome or disease; however, despite the field of biomarker research having established their important role in detecting allograft perturbations, they also have imperfections [63]. Neither marker is connected with molecular pathophysiology. Rather, each represents a consequence of substantial damage to the kidney, so there is a need for newer biomarkers, using proteomics or metabolomics, that enable an ability in the prediction of disease progression and the determination of therapeutic efficacy [60].

In the last part of our analysis, we tried to answer the initial question if the vasoactive, proinflammatory, or predictive properties of the examined modern biomarkers could translate into postoperative graft function based on classic kidney function parameters. The statistical analysis generated a vast amount of data since every concentration and its relatives was correlated between values obtained from reperfusion (Figure 4) with those from the seven-day observation period (Figure 5). There was a need to collect the above computations in a transparent way. After maintaining the number of correlations and leaving out individual statistical values, we show a summary in Table 2, where it is possible to better visualize which parameter could possibly play a role in the examined groups. It was unexpected that increased ET-1 concentrations seemed to positively influence further kidney function, especially in Group 1. ET-1, as a primarily vasoconstrictive agent, was expected to decrease the blood flow and, through that, diuresis and GFR. Nonetheless, we evaluated only 30 min of reperfusion, and ET-1 expression during the postoperative seven-day observation period remained unknown. We hypothesize that the vasodilatory ET_B_ receptors mentioned earlier could play a role in this process. We also cannot exclude the possibility that the initial short-term vasoconstrictive effect of ET-1 may act protectively against oxidative stress in reperfusion injury with subsequent vasodilation and better kidney function, but further investigation is needed. On the other hand, the vasoconstrictive characteristics of ET-2 and ET-3 were somewhat expressed. We expected to see more expressed correlation of IL-18, NGAL, and worse kidney function, but we realize that the IL-18 and NGAL production detected during reperfusion was just a start of a much broader period of time where IL-18 mediated immunologic reactions and NGAL participated in endothelial repairs.

### 4.6. Limitations

In our study design, we took advantage of a situation that for some time we had only one perfusion machine available before the other one was purchased. By default, one kidney was kept in HMP and the other was kept in SCS. This was an opportunity to compare those groups without deviating the transplantation protocol and unnecessary exclusion of HMP if both of them were been available.

The examined groups consisted of 22 patients each, which was possible according to perfusion machines status; however, group size was relatively too small and heterogeneous to extract all desired correlations from multifactorial analysis with a satisfactory precision. We chose to examine selected biomarkers according to their established role in IRI, but all of them were measured during reperfusion. We believe that this was a good choice for vasoactive agents such as endothelins, but we assume that it might not be the only period of their activity. Thirty minutes of reperfusion is a relatively narrow period, and it would be good to extend it by time points before KTx. Endothelins could be measured in the donor before procurement or in the recipient during preparation for surgical procedure. For IL-18 and NGAL, it has been proven that their secretion extends for a period much longer than a reperfusion itself, so we could think of time points from a postoperative hospitalization.

We chose to examine selected biomarkers in the blood. We could directly obtain material from the renal vein. It was our aim to choose a bloodstream that directly left the freshly re-perfused kidney that potentially contained accumulated examined biomarkers, but the clarification of the source would have required a comparison with peripheral blood, which was limited by the number of available ELISA assays. It was also possible to evaluate biomarkers in the urine, either selectively or for comparison. This was probably accurate, especially for IL-18 and NGAL because their dynamics one week after KTx correlate with DGF [48,64,65,66]. Histological evaluation would have been a valuable addition to our study; however, protocol biopsy is not a standard approach in Poland. It is rarely performed before transplantation. One exception is the need for the histologic confirmation of macroscopic findings after organ procurement. Quite rarely, the biopsy is performed after transplantation, except in cases of specific complications, e.g., acute rejection, but even then, it depends on severity or susceptibility for treatment.

## 5. Conclusions

Group 1 (hypothermic machine perfusion) was characterized by a tendency towards higher absolute concentrations of ET-1, IL-18, and NGAL, as well as lower concentrations of ET-2 and ET-3. Kidney reperfusion was associated with a tendency towards increasing concentrations of ET-1 and ET-2 and decreasing concentrations of IL-18 and NGAL in both examined groups. During the seven-day observation period, there was a statistically significant improvement in kidney function parameters in both examined groups, apart from a tendency towards higher GFR and diuresis in Group 1. There was an aggregation of statistically significant correlations between ET-1 and better kidney function parameters. There was an aggregation of statistically significant correlations between ET-2, IL-18, and worse kidney function parameters, which was more expressed in Group 1. All of the above tendencies, together with significant results, may speak for the hypothesis that there is a link between kidney function parameters and examined biomarkers. Moreover they may correspond with established beneficial effects of HMP.

## Figures and Tables

**Table 1:** Study group characteristics of recipients qualified for transplantation.

Recipients Characteristics (Unit)	Value Description	Group 1 HMP Group (*n* = 22)	Group 2 SCS Group (*n* = 22)	*p*
Age (years)	Median Range	55.0 34.0–69.0	54.0 33.0–70.0	0.36
Sex (*n*/%)	Men—M Women—F	15 (68.2%) 7 (31.8%)	11 (50%) 11 (50%)	0.22
BMI (kg/m^2^)	Median Range	27.3 23.2–28.1	27.9 24.1–30. 9	0.70
Hemodialysis time (months)	Median Range	37.0 18–53.5	48.0 24.5–60.0	0.43
Serum urea (mg/dl)	Median Range	101 75.0–140.0	85.5 55.0–111.0	0.19
Serum creatinine (mg/dl)	Median Range	7.4 5.6–9.4	7.9 6.6–8.6	0.99
eGFR (ml/min/1.73 m^2^)	Median Range	7.7 5.0–9.0	6.0 5.0–8.0	0.52
Leukocytes (G/μL)	Median Range	7.7 5.6–8.7	6.5 5.6–7.7	0.97
Platelets (G/μL)	Median Range	210.0 165.0–245.0	194.0 169.0–212.0	0.50
Potassium (mmol/L)	Median Range	4.5 4.2–4.7	4.6 4.6–4.7	0.40
Hematocrit (l/l)	Median Range	37.7 37.2–40.4	35.3 32.9–37	0.75
Mismatch A	Median Range	1 1–2	1 0–1	0.46
Mismatch B	Median Range	1 1–2	1 1–2	0.59
Mismatch DR	Median Range	1 0–1	1 1–1	0.50
HLA mismatches sum	Median Range	3 2–4	3 2–4	0.98
* HLA mismatch preferential points	Median Range	20 14–25	17 12–19	0.42
DGF	Incidence	*n* = 2 (9.1%)	*n* = 3 (13.6%)	0.60
CIT (h)	Median Range	18.4 4.5–26.9	11.6 4.9–22.9	0.07
Tac/CsA (%)	IS	95.5/4.5	95.5/4.5	NA
MMF (%)	IS	100	100	NA
Steroids (%)	IS	100	100	NA

Legend: BMI: body mass index; HLA: human leukocyte antigen *: national Poltransplant allocation rules (HLA-A(+2 preferential points per 1 matched A antigen), -B(+5 points per 1 B antigen respectively), and -DR(+10 points per 1 DR)); DGF: delayed graft function; CIT: cold ischemic time; IS: immunosuppression; Tac: tacrolimus; CsA: cyclosporine; MMF: mycophenolate mofetil; eGFR: estimate of the glomerular filtration rate.

**Table 2:** Summary of correlation matrix (ET-1, ET-2, ET-3, IL-18, and NGAL) vs. (D—diuresis; Cr—creatinine; Ur—urea; GFR—glomerular filtration rate; K—potassium) in storage groups (red gradient—positive impact; blue gradient—negative impact; more intensive color—higher number of statistically significant correlations; ◯—positive correlation; ⚫—negative correlation; *, **, #, ##, +, ++—discussed aggregations).

	Group 1 (HMP)								Group 2 (SCS)							
	D	Cr	Ur		GFR		K		D	Cr	Ur		GFR		K	
	abs	abs	abs	Δ	abs	Δ	abs	Δ	abs	abs	abs	Δ	abs	Δ	abs	Δ
ET-1 1 m *									◯	⚫	⚫	⚫	◯	◯		
ET-1 30 m **	◯	⚫	⚫		◯		⚫									
ET-1 Δ ^∗∗^	◯	⚫		⚫	◯											
ET-2 1 m		◯			⚫			◯								
ET-2 30 m ^#^		◯	◯	◯	⚫	⚫	◯	◯	⚫	◯	◯		⚫	⚫		
ET-2 Δ			◯												◯	◯
ET-3 1 m		◯			⚫											
ET-3 30 m ^##^							◯		⚫	◯	◯		⚫			
ET-3 Δ		⚫			◯											
IL-18 1 m	◯						⚫								◯	
IL-18 30 m	◯														◯	
IL-18 Δ **^+^**	⚫	◯	◯	◯	⚫		⚫	⚫			◯					⚫
NGAL 1 m **^++^**		◯			⚫											
NGAL 30 m **^++^**					⚫		◯									⚫
